# Psychological Benefits of Arts Participation for Emerging Adulthood: A Pathway to Flourishing

**DOI:** 10.3390/bs14060448

**Published:** 2024-05-26

**Authors:** Jinming Fan, Xiaoli Ni, Ting Wu, Yidi Wang, Yuyan Qian

**Affiliations:** 1 Institute of Social Psychology, School of Humanities and Social Science, Xi’an Jiaotong University, Xi’an 710049, China; fjm2018@stu.xjtu.edu.cn (J.F.); nixiaoli@xjtu.edu.cn (X.N.); 36001@xacom.edu.cn (T.W.); juliaWYD@outlook.com (Y.W.); 2 Northwest National Music Research Center, Xi’an Conservatory of Music, Xi’an 710061, China

**Keywords:** arts participation, positive psychology, flourishing, flow, aesthetic emotion, ego identity

## Abstract

This study examined 430 Chinese college students’ engagement in arts activities and the psychological benefits derived from such activities. The research differentiated between various types of arts participation and ways of involvement and examined four potential positive psychological outcomes. The findings revealed correlations between (1) creative participation in the performing arts, ‘flow’, and aesthetic emotions; (2) consumptive participation in the visual arts and aesthetic emotions; and (3) creative participation in the literary arts and ego identity. Holistic arts participation demonstrated a significantly positive relationship with flourishing. A path analysis showed that flow experience and aesthetic emotions served as mediators in the mechanism through which holistic arts participation affected flourishing, with a chained mediation effect from flow experience to ego identity. This study confirms that arts participation is an effective pathway for individual flourishing and that more diverse and profound engagement in the arts can lead to sustained and widespread happiness.

## 1. Introduction

Throughout history, the importance of art for achieving comprehensive human development has been indisputable [1,2]. However, the literature has a limited understanding of whether the psychological benefits of artistic engagement vary due to the different art forms and operationalization approaches employed, and the pathways through which arts participation contributes to human well-being are still not well explored. This study focused on the associations between various types of arts participation and operationalization approaches with positive psychological indicators across different domains. The findings can provide theoretical support for the development of art education and public health initiatives.

### 1.1. Types and Operationalization of Artistic Participation

The arts include several forms with rich connotations and contents. There has been no consensus in academia on the definition of arts engagement. However, scholars generally agree that the form and the way of arts engagement are crucial to defining arts engagement. For example, Davies et al. [3] indicate that arts engagement can be defined by art forms, activities, and levels of participation, with forms including five categories: (1) Performing arts; (2) Visual arts, design, and crafts; (3) Community or cultural festivals, fairs, and events; (4) Literature; and (5) Online, digital, and electronic arts [3]. The article provides 91 different arts activities based on the levels of art form and participation, laying the foundation for research in the field of arts and health. Sonke et al. [4] broadly and inclusively define the concept of arts engagement through two segments: participation ways and art forms, encompassing various activities such as creation, positive experiences, and observing art. They note that the ways people engage in arts include roles such as creators, collaborators, audiences, and observers, and art forms include dance or movement, literary arts, media, music, drama or performance, visual arts, crafts, design, and more.

In their empirical study, Kou et al. [5] provided a concise definition of arts engagement, categorizing it into two types: creative engagement and consumptive engagement. Creative participation includes arts activities such as painting, playing musical instruments, performing, and dancing. Consumptive participation includes activities such as visiting art museums, attending performances, and purchasing artworks. They also considered three major art forms: visual arts (e.g., painting, sculpture, and design), performing arts (e.g., music, dance, and drama), and literary arts (e.g., poetry, prose, and novels). Their study indicates that engaging in artistic creation and consumption can enhance aesthetic enjoyment and contribute to overall well-being.

Empirical research has two approaches for the operationalization of artistic participation. The first distinguishes between the form and content of arts participation, while the second integrates arts participation from different art categories into a single indicator known as ‘combined arts participation’ or something similar, using the total score of arts participation in research without differentiating between art forms. Both research methods are useful and can provide diverse information. Examining the different types of arts participation can provide more ample and specific information for research, while studying the arts comprehensively can offer guidance for more ‘general’ policy formulations [6].

### 1.2. Potential Benefits of Arts Engagement

Art has always been embedded in daily life; however, it is only recently that the psychological benefits of arts engagement are being examined in psychology. Studies have extensively explored whether and how art impacts individuals, proposing numerous theoretical models. For instance, influenced by positive psychology, Lomas [7] introduced the concept of ‘Positive Art’ to suggest that engagement in visual arts, music, literature, drama, and other arts activities can bring about five positive outcomes for individuals: sense-making (finding meaning in life), enriching experiences (feeling emotions), aesthetic appreciation (joy from the beauty of the work), entertainment, and bonding with others. Tay et al. [2] offered another delineation of the psychological benefits of arts engagement into four categories: (1) instant positive changes in neurology, physiology, and emotion (e.g., regulating emotions, relieving stress); (2) enduring psychological competencies (e.g., enhancing self-efficacy, creativity, adaptability); (3) general well-being indices (e.g., enhancing hedonic or eudaimonic happiness and physical health); and (4) positive changes in normative developmental outcomes (e.g., fostering the formation of important values like freedom and justice, altruistic behavior, and civic engagement). The model hypothesized that the impact of arts engagement on individuals varied depending on the type of arts engagement.

Building upon these theoretical models, this study adopts an empirical analysis approach to investigate the relationships between arts engagement (particular and holistic) and different domains of positive psychological outcomes. Specifically, this study focuses on four categories of positive psychological outcomes: (1) flow, (2) aesthetic emotions, (3) ego identity, and (4) flourishing.

Flow is a state of profound spiritual enjoyment characterized by complete immersion in the present moment and a transient loss of awareness of the self and time. It typically occurs during activities that are challenging but aligned with an individual’s skill level [8]. Engaging in arts activities is one significant way to induce a flow experience. A longitudinal study of New Zealand high school students demonstrated that participation in performing arts activities facilitated flow among the participants [9]. A qualitative study focusing on artistically gifted adolescents in Singapore captured elements associated with flow, such as enjoying the activity, a strong sense of focus, and clear goals, as the adolescents shared their experiences of various art forms, including dancing, painting, music, and drama [10]. Another experiment involving Canadian university students found that dancing to music significantly increased the occurrence of flow [11]. Visiting museums or art galleries can create flow experiences [12], especially when using augmented reality guides [13].

Aesthetic emotions comprise an intricate complex of multiple emotional components at various levels, regulated by individual aesthetic preferences and judgments [14]. To date, the academic community has not reached a consensus on the specific categories of aesthetic emotions. However, a recent study has suggested that awe, being moved, and wonder are three typical and uncontroversial aesthetic emotions [15]. Arts engagement is a crucial pathway for inducing aesthetic emotions. Empirical studies indicate that symphonic music, painting, and films are common elicitors of awe-related emotions [16]; watching films can evoke the emotional experience of being moved [17]; and some great artworks can activate a sense of wonder [18].

Erikson [19] identified the acquisition of ego identity as the primary developmental task in adolescence. During adolescence, individuals ponder who they are, where they are headed, and their place in society. Answering these questions entails individual self-evaluation and positioning regarding career, politics, religion, and values [19]. As society advances, diverse evidence indicates that the exploration of ego identity among young adults during the period of emerging adulthood (i.e., between the ages of 18 and 25) is more pronounced than during adolescence [20]. This cohort of individuals has exited adolescence but not yet fully entered adulthood, where individuals assume comprehensive responsibility. The trajectory of their futures remains undetermined, necessitating an examination of a multitude of life possibilities in domains such as romance, vocation, and worldview to ascertain their ego identity. Ego identity is gradually formed through interactions between individuals and their environments [21]. Engaging in arts activities provides emerging adults with a unique environment in which to present and explore their ego identities. Pelowski and Akiba [22] suggest that individuals carry fundamental meanings about themselves, others, objects, or behaviors when engaging in artistic activities, such as ‘Who am I?’ ‘What is art?’ ‘What is the relationship between art and me?’ that collectively form what they refer to as the ‘ideal self-image’. Empirical studies suggest that participating in performing arts workshops can enhance the ego identity of first-year university students [23] while offering art-based courses can strengthen the integration of ego identity among emerging adults [24]. Participating in arts activities and having an artistic identity (such as being an art enthusiast or artist) help individuals develop a more intimate and comprehensive self-awareness, forming a more positive self-image [25,26]. Clinical research has also found individuals with depression can express themselves, experience emotional resonance, and thus develop an ego identity through reading [27].

Flourishing is an emerging concept in the field of positive psychology that is used to measure the optimal state of an individual’s psychological functioning [28]. It emphasizes the individual’s positive mental health and capacity to function well in life [29]. Flourishing is considered an overarching term and a multidimensional construct that encompasses a range of positive states and outcomes related to a person’s thriving, such as positive emotions, life satisfaction, meaning, social relationships, personalities and virtues, and health [30]. Flourishing is closely related to the concept of well-being; however, it broadly ensures an individual’s healthy development. Arts participation involves sensory, cognitive, emotional, social, and physiological processes, among others, that help individuals reach a state of flourishing [31]. For example, visiting art museums can yield positive outcomes related to flourishing [32]. One survey indicated a longitudinal connection between arts participation and subsequent levels of flourishing in young adults; that is, longer engagement in arts activities leads to higher levels of flourishing [33]. An intervention study also suggested a significant increase in overall happiness reported by participants after three months of weekly collective music performance [34].

### 1.3. Psychological Processes Model of Arts Engagement’s Influence on Flourishing

Despite the widespread recognition of the psychological benefits of arts engagement, existing research has not fully clarified how arts engagement influences flourishing. Seligman’s [35] PERMA model suggests that individuals achieve enduring well-being or psychological flourishing when they experience positive emotions (P), engage and enjoy activities they are interested in (E), maintain healthy relationships (R), have clarity in meaning and purpose in life (M), and accomplish personal goals (A). This model provides a foundation for exploring the pathways through which arts participation influences flourishing.

Studies [2,36] have identified five mechanisms through which arts engagement can influence flourishing: reflection, acquisition, immersion, socialization, and expression (collectively referred to as RAISE). Reflection involves the internal motivation to explore or transform one’s identity, values, and beliefs. Acquisition pertains to the acquisition or development of skills. Immersion refers to the experience of high interest and absorption. Socialization involves social connections through arts engagement. Expression involves expressing oneself through arts activities. In essence, engaging in arts activities can provide rich, immersive experiences that allow individuals to socialize, reflect, and express themselves, thereby enhancing their well-being and equipping them with skills that contribute to their flourishing.

Empirical evidence suggests that flow experiences mediate the relationship between arts engagement and well-being [37,38]. The more individuals are immersed in the flow experience brought by the arts, the more likely they are to trigger dialectical thinking about themselves [39], promoting the development of ego identity [40]. The aesthetic emotions, such as awe, wonder, and being moved, generated during arts engagement enhance well-being [41,42], rectify the mind, prompt reflection, and facilitate ego identity [43]. Ego identity is a state associated with high psychological well-being [44,45] and can create a sustainable state of positive living [46].

### 1.4. Literature Gaps

Despite the valuable results of previous studies on the psychological benefits of art, the overall state of research is still in the exploratory phase, leaving numerous gaps to be filled. For instance, existing research often considers artistic engagement as a whole, and several empirical studies that individually examine the forms and content of artistic engagement (e.g., Kou et al., [5]) lack datasets that simultaneously include various art forms. Hence, they can only use multiple datasets that each contain only one art form, leading to inconsistencies in data availability across the datasets and preventing the research results from presenting a unified pattern across the different datasets. There has been no exploration of how different operationalization approaches toward arts engagement may impact positive psychological outcomes. Furthermore, many empirical studies typically select a single index for positive psychological outcomes, such as prosocial behavior, with limited attention to multiple indices. Few studies have incorporated various indicators of human well-being [38]. Primarily focused on highly skilled professional artists, they lack clarity in application to more general populations, especially to the emerging adulthood group who are in a crucial period of ego identity formation. Moreover, the existing research concentrates on qualitative explanations and theoretical hypotheses regarding the pathways through which artistic engagement influences human well-being. Few empirical studies have tested these theories or investigated the intrinsic relationship between different domains of psychological benefits.

### 1.5. The Present Research

This study aimed to explore the relationship between the approaches of operationalization on artistic engagement and various positive psychological outcomes. Specifically, this research addresses three questions. First, what is the relationship between different types of artistic engagement (creation and consumption in the visual, performing, and literary arts) with the variables of positive psychology (flow, aesthetic emotions, ego identity, and flourishing)? Based on existing research, this paper preliminarily assumes that the psychological benefits of artistic engagement will vary with different types of artistic engagement and psychological outcomes (H1). As this is an exploratory study, no specific assumptions about the patterns of differentiation have been made. Second, what is the influence of artistic engagement as a whole on positive psychological outcomes among university students? Based on previous research, this paper hypothesizes that comprehensive artistic engagement is positively correlated with positive psychological outcomes among university students (H2). Third, through what pathway does arts engagement as a whole influence flourishing? Considering previous research, this study proposes a hypothetical model to describe the relationships between key variables (artistic engagement, flow, aesthetic emotions, ego identity, and flourishing). We hypothesize that (1) artistic engagement has a significantly positive relationship with flourishing (H3a); (2) flow (H3b), aesthetic experience (H3c), and ego identity (H3d) mediate the relationship between arts engagement and flourishing; and (3) flow and ego identity (H3e) and aesthetic emotions and ego identity (H3f) play chain-mediating roles in the relationship between artistic engagement and flourishing.

## 2. Materials and Methods

### 2.1. Participants and Procedure

The research participants include a group of Chinese university students. Over the past half-century, with the rapid development of Chinese society, the marriage and childbearing age of the younger Chinese generation has been universally postponed to after the age of 25 [47], and the time spent studying in school has also increased. The university period is marked by individual exploration and instability, self-focus, and various possibilities [48]. Additionally, Chinese aesthetics has always pursued the aesthetic interest of ‘the unity of heaven and man’, emphasizing the achievement of optimal harmonious coexistence between humans, nature, spirit, and the universe in aesthetic experiences and the realization of a good life [49]. Recently, the Chinese government has continuously advocated for the strengthening of aesthetic education in higher education institutions [50], making efforts to enable most students to participate in school art practice activities, thus establishing opportunities and platforms for university student arts participation. Therefore, the psychological benefits of Chinese university students’ arts participation must be assessed.

A simple random cluster sampling method was employed to conduct a questionnaire survey of Chinese university students who had participated in arts activities within the past year. The survey was conducted offline, with a total of 450 paper–pencil questionnaires distributed, and after excluding data with missing values and invalid responses (such as excessive unanswered questions, patterns in selected options, or samples indicating no arts participation in the past year), 430 valid questionnaires were obtained, with an effective response rate of 95.56%. Of the participants, 282 were female (65.6%), and 148 were male (34.4%). The age range of the sample was 17–25 years (M = 20.16, SD = 1.58). The study was approved by the Ethics Committee of Xi’an Jiaotong University (No. 20221435). Before participating in the formal questionnaire survey, all participants signed an informed consent form and received a gift worth five yuan as compensation upon completing the questionnaire.

### 2.2. Instruments

#### 2.2.1. Arts Participation

Drawing on Kou et al. [5], this study measured the extent of arts participation through six questions. Participants were asked whether they had engaged in the following arts activities in the preceding year: (1) any visual arts creation activities, such as drawing, pottery, sculpture, or crafts; (2) any performing arts creation activities, such as performing in plays, dancing, or playing music; (3) any literary arts creation activities, such as writing novels, poetry, or prose; (4) any visual arts consumption activities, such as visiting art exhibitions, art museums, or purchasing artworks; (5) any performing arts consumption activities, such as watching films, plays, concerts, or dance performances; and (6) any literary arts consumption activities, such as reading novels, poetry, or prose. All items were answered as either yes or no, with yes = 1 and no = 0.

In subsequent analyses, arts participation was input as a single item into the analytical model to measure the relationship between different types of arts participation and positive psychological outcomes; it was summed across all items to provide an overall structure entering the model to assess the pathways through which arts participation influenced flourishing. The method of aggregating various forms of arts participation to replace the individual degree of arts participation is commonly employed in research exploring the relationship between art and the development of positive psychological outcomes [31]. In this study, the total score of the six items represents the diversity of arts participation, with a higher score indicating a broader range of arts participation.

#### 2.2.2. Flow in Arts Participation

The Flow Short Scale [51] was employed to measure individuals’ general flow experiences in all types of arts participation without distinguishing between different arts activities over the past year. This scale consists of ten items, with four items measuring immersion (e.g., ‘During arts activities, I did not notice the passage of time’) and six items measuring fluency (e.g., ‘During arts activities, my thoughts or activities run fluidly and smoothly’). These were rated using a 7-point Likert scale (1 = strongly disagree, 7 = strongly agree). The total score was used, with higher scores indicating a higher level of flow experience during arts participation. Previous research has indicated the good reliability and validity of this scale in measuring flow in college students’ arts participation [52] and has been used in Chinese culture [53]. In this study, the internal consistency coefficient of the scale was 0.95.

#### 2.2.3. Aesthetic Emotion in Artistic Participation

Aesthetic emotions were measured using three subscales, including awe, being moved, and wonder, that were extracted from the AESTHEMOS Scale [54]. In this study, the scale was used to measure the intensity of aesthetic emotions that individuals experienced in all types of arts participation without distinguishing between different arts activities over the past year. The questionnaire, derived from these subscales, comprised six items, with two items measuring awe (‘I feel awe’ and ‘I find it sublime’), two items measuring being moved (‘I am deeply moved’ and ‘It touches me’), and two items measuring wonder (‘This feels wonderful’ and ‘It intoxicates my heart’). Participants used a 5-point Likert scale (1 = not at all, 5 = very much) for each item, and the total score was calculated, with a higher score indicating a stronger aesthetic emotion during arts participation. Previous studies show that the scale has good reliability and validity [55] and is applicable to Chinese college students [56]. The internal consistency reliability in this study was 0.93.

#### 2.2.4. Ego Identity

The Ego Identity Scale [57] was used to assess individuals’ overall evaluation of their ego-identity status. This scale conceptualizes ego identity as a continuum from ego-identity diffusion to ego-identity achievement and utilizes a forced-choice format to minimize the impact of social desirability effects. The scale comprises 12 items, each presenting two sentences describing ego identity—one indicating ego-identity achievement (e.g., ‘Even when I seem to be the only one in a group holding a particular view, I always express my opinion’), and the other indicating ego-identity diffusion (e.g., ‘When I seem to be the only one in a group holding a particular view, I try to remain silent and avoid feeling nervous’). Participants were instructed to assign a score of 1 if they chose the sentence representing ego-identity achievement and 0 if they chose the sentence representing ego-identity diffusion. The total score was obtained by summing all items, with a higher score indicating a higher level of ego-identity achievement. Studies have shown that the scale is suitable for college student populations [58], and the Chinese version of the scale, after back-translation, is applicable to the Chinese cultural context [59].

#### 2.2.5. Flourishing

The Short Flourishing Scale [28] was used to measure individual well-being in the general life domain. This scale comprises eight items (e.g., ‘My life has a clear sense of purpose and meaning’), describing human functioning from eight aspects: competence, engagement, meaning and purpose, optimism, self-acceptance, supportive relationships, the well-being of others, and being respected. It was rated on a seven-point Likert scale (1 = strongly disagree, 7 = strongly agree). The total score, derived by summing the responses, reflected an individual’s possession of positive psychological resources and social functioning. A higher score indicated a stronger sense of flourishing as perceived in the general life domain. Previous research has demonstrated the Chinese version of the Flourishing Scale has good reliability and validity [60] and has been widely applied to college student populations [61,62]. The internal consistency reliability in this study was 0.94.

#### 2.2.6. Control Variables

Previous research has indicated that the psychological benefits of arts participation can be influenced by gender, age [63], family economic status, and prior art-learning experiences [1,4]. Therefore, in addition to standard demographic variables, such as gender and age, this study measured participants’ subjective family economic status and prior art-learning experiences as control variables. Sex was treated as a categorical variable (1 = male, 0 = female). Age was treated as a continuous variable obtained by asking participants, ‘What is your age?’ Prior art-learning experience was a categorical variable assessed by asking participants, ‘Have you had art-learning or practice experiences lasting six months or more (such as taking courses or classes in music, visual arts, performing arts, dance, calligraphy, literature, etc.)?’ (1 = yes, 0 = no). Subjective family economic status was measured using the MacArthur Scale of Subjective Social Status [64], in which participants were presented with a ladder diagram depicting ten rungs and asked to position themselves on the ladder based on their perceived income, education, and occupational circumstances within their family. A rating of 1 indicated the lowest social status, whereas 10 indicated the highest. This scale has demonstrated good reliability and validity and has been widely used in empirical research [65].

### 2.3. Data Analysis

In this study, we used SPSS 25.0 to analyze the collected data. First, we conducted descriptive statistical analyses to examine the current state of arts participation. Second, we used multiple regression analysis to explore the relationships between the two operationalizations of arts engagement (distinguishing versus not distinguishing between arts activities) and positive psychological outcome variables, with flow, aesthetic emotions, ego identity, and flourishing as dependent variables, and arts engagement as the key independent variable, while controlling for gender, age, family economic status, and prior art-learning experiences. To examine the mediating effects, we employed the Hayes bootstrapping method and analyzed using the Process tool in SPSS [66]. The advantage of the bootstrapping method is its ability to assess multiple mediating variables by isolating specific mediating routes and comparing the differential impacts of various mediation routes [67]. This study employed Model 80 from the Hayes Process plugin, aligning with the hypotheses posited in this study, for a comprehensive examination of multiple mediations.

## 3. Results

### 3.1. Relationship between Different Types of Arts Engagement and Positive Psychological Outcomes 

The descriptive statistical analysis shows that in the past year, 41.2% of college students have participated in visual arts creation activities, 25.1% have participated in performing arts creation activities, and 32.8% have participated in literary arts creation activities. Additionally, 57% of college students have engaged in visual arts consumption activities, 70.5% have participated in performing arts consumption activities, and 82.3% have participated in literary arts consumption activities. Furthermore, 75.6% of college students have had experience with art study for at least six months. The subjective family economic status ranges from 1 to 10, with an average of 5.62 and a standard deviation of 1.74.

Table 1 presents the regression models for the different types of arts participation and positive psychological outcomes. The results indicate that controlling for gender, age, subjective family economic status, and prior art-learning experiences, individuals engaged in performing arts creation activities scored significantly higher in the experience of flow compared to those who did not participate in such activities (*β* = 0.09, *p* < 0.05). Individuals engaged in performing arts creation activities also scored significantly higher in aesthetic emotions than their non-participating counterparts (*β* = 0.12, *p* < 0.01). Those who participated in visual arts consumption activities scored significantly higher in aesthetic emotions than those who did not engage in visual arts consumption activities (*β* = 0.14, *p* < 0.01). Individuals engaged in literary arts creation activities scored significantly higher in ego identity than those who did not participate in literary arts creation activities (*β* = 0.12, *p* < 0.05). However, no form of arts participation was significantly related to flourishing. These results indicate that the psychological benefits of arts participation vary with different artistic activities, supporting H1.

Regarding the control variables, female students reported more flow experiences in artistic activities (*β* = −0.11, *p* < 0.05) and stronger aesthetic emotions (*β* = −0.10, *p* < 0.05). Age was negatively correlated with aesthetic emotions, with older individuals experiencing a lower intensity of aesthetic emotions in artistic activities (*β* = −0.12, *p* < 0.05). Higher subjective family socioeconomic status was associated with more flow experiences in artistic activities (*β* = 0.11, *p* < 0.05), as well as higher levels of ego identity (*β* = 0.21, *p* < 0.001) and flourishing (*β* = 0.20, *p* < 0.001). Individuals with six months or more of art-learning experience gained more flow experiences (*β* = 0.24, *p* < 0.001) and stronger aesthetic emotions (*β* = 0.18, *p* < 0.001) in artistic activities.

### 3.2. Relationship between Comprehensive Arts Engagement and Positive Psychological Outcomes

Table 2 presents the regression models for total arts participation scores and positive psychological variables. The results indicate that, after controlling for gender, age, subjective family economic status, and prior artistic learning experiences, the diversity of arts engagement was significantly related to flow experience (*β* = 0.17, *p* < 0.001) and aesthetic emotions (*β* = 0.19, *p* < 0.001). It also explained a significant amount of the variance in ego identity (*β* = 0.13, *p* < 0.01) and flourishing (*β* = 0.10, *p* < 0.05). Therefore, H2 was supported. The relationships between the control variables and the positive psychological outcome variables are similar to those found in Table 1.

### 3.3. Relationship between Combined Arts Engagement and Flourishing

To analyze the mechanisms through which arts participation relates to flourishing and explore the impact of multiple mediating paths, including short-term flow experience, aesthetic emotions, and general psychological capacities such as ego identity, this study employed the Hayes bootstrapping method to test the mediation effects using the Process tool in SPSS. The results are shown in Table 3.

Model 1 represents the baseline regression results. In Model 2, the regression of arts participation’s total score on flow showed a significant positive relationship (*β* = 0.17, *p* < 0.001). Model 3, depicting the regression of arts participation on aesthetic emotions, showed a significant positive relationship (*β* = 0.19, *p* < 0.001). Model 4, presenting the simultaneous regression of arts participation, flow, and aesthetic emotions on ego identity, indicated a significant positive relationship between flow and ego identity (*β* = 0.31, *p* < 0.001), whereas arts participation and aesthetic emotions were not significantly associated with ego identity. Model 5, displaying the regression results for arts participation, flow experience, aesthetic emotions, and ego identity on flourishing, revealed that flow experience (*β* = 0.14, *p* < 0.01), aesthetic emotions (*β* = 0.22, *p* < 0.001), and ego identity (*β* = 0.50, *p* < 0.001) all had a significant positive relationship with flourishing.

**Figure 1:**
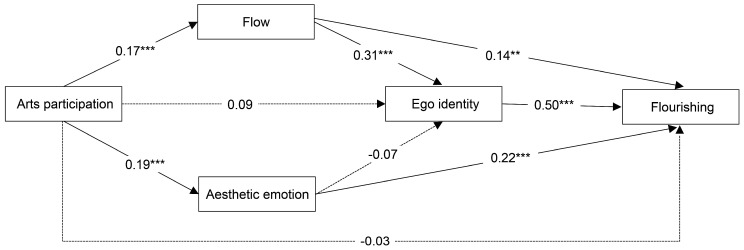
Multiple mediation test diagram. ** *p* < 0.01, *** *p* < 0.001.

These regression models suggest the presence of multiple-chain mediation effects. To ensure the reliability of the results, bootstrap tests were conducted for each mediating path, as shown in Table 4. Except for the two paths, ‘arts participation—ego identity—flourishing’ and ‘arts participation—aesthetic emotions—ego identity—flourishing’, which were not significant, all other mediating paths were significant. Therefore, H3b, H3c, and H3e were supported, whereas H3a, H3d, and H3f were not. The model diagram is presented in Figure 1.

## 4. Discussion

By distinguishing between six types of arts participation and four positive psychological indicators, this study comprehensively examined the psychological benefits and pathways of arts participation, representing an innovative approach. The findings indicate that the psychological benefits of arts participation vary depending on the type of art and the operationalization approaches employed. It also showed the correlations between performing arts creation and flow experience; aesthetic emotions, visual arts consumption, and aesthetic emotions; and literary arts creation and ego identity. Moreover, the findings revealed that no singular form of arts participation significantly affected flourishing. However, when considered as a whole, arts participation has a significant and positive relationship with flourishing, suggesting that more diverse and profound engagement in the arts leads to more enduring and widespread well-being. The path analysis further indicated that in the mechanism through which combined arts participation affected flourishing, flow experience and aesthetic emotions served as mediators, with a chained mediation effect from flow experience to ego identity. 

### 4.1. Relationship between Types of Arts Participation and Positive Psychological Outcomes

This study revealed that performing arts creation activities (such as drama, dance, and musical performances) are associated with a strong sense of flow, aligning with previous research suggesting that involvement in performing arts creation could enhance flow [9]. Flow is characterized by intense concentration and immersion accompanied by pleasure, satisfaction, and a loss of awareness of time and space [8]. The unique aspect of the performing arts is their ability to opportune participants to become ‘immersed’ in the performance. This diminishes the psychological distance between the performer and the arts activity, naturally engrossing them in the performing arts, thereby increasing the likelihood of experiencing flow. In addition, the performing arts exhibit stronger social interactivity than other art forms. Research suggests that social interaction is a pathway for enhancing flow [68]. During performances or rehearsals, participants may find like-minded friends, and the resulting resonance and sense of belonging may enhance the emotional connections between individuals and arts activities, thereby deepening their experience of flow.

Regarding aesthetic emotions, this study found that individuals who engaged in performing arts creation (such as drama, dance, or musical performances) or visual arts consumption (such as visiting art museums or purchasing artworks) experienced more intense aesthetic emotions than others did. Other studies have indicated that the generation of aesthetic emotions is linked to an understanding of the meaning of art. The greater the participants’ understanding of arts activities, the stronger their aesthetic emotions [69]. Creators and performers who craft vivid and lively artistic images demonstrate their understanding of art and elicit aesthetic and emotional experiences [70]. Relevant studies have suggested that experiencing aesthetic beauty and seeking emotional resonance are crucial reasons for visiting art museums [71]. Visitors to art museums may experience awe as a result of their visits [72]; the impact of artworks can transcend their temporal and spatial limitations, allowing individuals to detach themselves from the mundane aspects of daily life and enter a transcendent aesthetic space [1]), thereby experiencing stronger inspiration, admiration, and awe.

This study found that engaging in literary arts creation (writing novels, poems, and prose) was associated with a higher level of ego identity achievement. Previous research has suggested that writing is an effective means of achieving ego identity [73,74]. Compared to art forms like reading, literary creation has a greater relationship with individual ego identity. This may be because literary creation requires stronger reflectiveness. When individuals hold a pen for literary creation, the fundamental questions revealed behind the act are related to each writer’s fundamental reflections on life and the self. Ego identity is a process of reflective self-understanding [75], and the reflectiveness of literary creation naturally aids individuals in constructing a subjective identity, thus promoting the formation of ego identity [76].

Furthermore, this study found that prior art-learning experience is positively correlated with the flow experience and aesthetic emotions obtained in arts activities, which is consistent with existing research. Previous studies have shown that art education not only cultivates artistic talent and taste but also produces individuals with strong aesthetic sensitivity [43], and individuals who are more familiar with the art knowledge system may have stronger aesthetic experiences [77]. Compared to individuals without art training, those with prior art-learning experience have better opportunities to appreciate art, are more familiar with the art knowledge system, may possess higher perceptual abilities in art, and may easily achieve positive experiences such as flow in arts activities. Regarding demographic variables, this study found that females have stronger flow experience and aesthetic emotions in arts activities than males. Related research suggests that compared to males, females participate in more arts activities [78] and are more inclined to regulate their emotions through arts engagement [79]. The positive correlation between subjective family economic status and positive outcomes has also been validated [63]. Regarding age, this study found that as the age of the university student group increases, the intensity of aesthetic emotions, such as awe, obtained in arts activities decreases, prompting further consideration. Notably, the age variability in the university student group in this study is relatively low. Future studies must investigate diverse ages to understand better the differences in psychological benefits obtained from arts participation among different age groups.

### 4.2. Relationship between Operationalization Approaches of Arts Participation and Positive Psychological Outcomes

This study employed two approaches to the concept of arts participation, revealing the distinct relationships between different types of combined arts participation and positive psychological outcomes. This supports the empirical view that differing operational definitions of core variables can lead to disparate conclusions [80]. Specifically, this study found variations in the relationship of the forms of arts participation on flow, aesthetic emotions, and ego identity. However, concerning flourishing, engagement in a single form of arts participation showed no significant effect, whereas overall, arts participation demonstrated a significant positive relationship. This suggests that for the general life well-being index, the specific type or content of arts participation may not be crucial; instead, the breadth and depth of engagement may be influential factors. This aligns with the theoretical viewpoint that more diverse and profound engagement leads to enduring and widespread well-being [2]. Therefore, when designing art intervention programs or formulating art education initiatives, there should be a focus on providing participants with more diverse, sustained, and profound arts experiences and transforming arts participation into a lifestyle habit.

Furthermore, the lack of any consistent pattern of psychological outcomes across different types of arts engagement prompts future studies to understand the contribution of various types of arts engagement to well-being. Existing empirical research on the psychological benefits of arts engagement primarily focuses on creative activities, with relatively less attention given to consumptive engagement. Future studies must explore whether different arts activities have different pathways to flourishing and whether different forms of arts engagement can be aggregated into a single score.

### 4.3. Mediating Pathways of Arts Participation Affecting Flourishing

The results of the path analysis indicated that the direct path from overall arts participation to flourishing was not significant. However, it could exert a relationship through chains of mediating factors, including ‘arts participation—flow—flourishing’, ‘arts participation—aesthetic emotions—flourishing’, and ‘arts participation—flow—ego identity—flourishing’. This finding underscores the significance of positive experiences in arts participation and supports the view that engaging in and enjoying everyday activities are crucial pathways to achieving a fulfilling life [81,82]. This finding highlights the unique psychological benefits of arts engagement for university students. For individuals in emerging adulthood, arts engagement provides opportunities to explore various themes related to ego identity. This aligns with the findings of existing research [43]. When individuals immerse themselves in arts experiences, their identity naturally becomes stimulated, forming a more mature sense of identity through continuous exploration and ultimately leading to psychological prosperity. Additionally, this finding also suggests that in artistic interventions or education, greater emphasis should be placed on the inherent emotional and spiritual values of art. Participants should be consciously guided to focus on the present moment, experience the aesthetic emotions inherent in the artwork, feel the connections between the artwork and social realities, and engage in reflective self-examination. Notably, compared to other rewarding experiences, aesthetic experiences are universal and relatively independent [83]. Therefore, for individuals with limited access to other positive experiences (such as social or economic rewards), cultivating their ability to participate in and enjoy arts activities may be more beneficial.

Notably, the measurement of arts engagement in this study, wherein respondents were asked whether they had participated in six types of arts activities in the past year, effectively distinguishes participants from non-participants but does not capture the frequency or intensity of the respondents’ engagement with arts activities. In other words, we could not distinguish between individuals who may have only attended a live theater once in the past year and those who attend more frequently. Frequent or intensive participation may be more significantly related to positive psychological outcomes. Regarding this, the overall measure of arts engagement generated by summing up the six items does not accurately measure the depth of engagement with art but merely reflects the diversity of arts engagement. Whether the mediating paths discovered in this study would depend on the different types of arts activities also needs to be tested. Furthermore, considering the reality of the increasing popularity of art education in Chinese universities, this study excluded samples that had not participated in any arts activities in the past year, which may affect the results’ reliability. Regarding the selection of mediating variables, this study chose ‘positive art’ variables such as flow and aesthetic emotions; however, experiences in arts engagement may not always be positive. Future studies must consider potential negative experiences that may arise from arts engagement. In summary, future research should refine the operationalization of the ‘arts engagement’ variable to obtain more reliable research conclusions.

### 4.4. Limitations and Future Directions

This study has certain limitations. First, although six types of arts participation were differentiated based on both the form of art and level of engagement, specific details of artwork (such as comedy or tragedy), the purpose of arts participation (e.g., educational or recreational), or the channels through which arts participation occurs (such as traditional in-person participation or online participation) were not considered. In future research, a more nuanced definition and measurement of the core variable of arts participation should be pursued to achieve conclusions of greater depth and comprehensiveness. Second, while two operationalization approaches of arts participation were compared, noting more diverse and in-depth arts participation may provide enduring and widespread well-being, the relationship between variables such as the time, frequency, and intensity of arts participation and well-being in general life domains were not examined. Future studies must examine this. Third, the conclusions drawn from a survey of Chinese university students may not be generalizable. For better generalizability, future studies must consider diverse populations, such as artists, specific clinical samples, or individuals from diverse cultural backgrounds. Finally, a cross-sectional research design was employed, and all findings were essentially correlational, with unclear causal relationships among the variables. The study relied on retrospective questionnaire measurements for all variables and did not directly explore highly situational factors, such as flow and aesthetic emotions, during specific arts activities. Building upon this study’s findings, future research could use longitudinal or experimental designs to analyze further the causal relationships between arts participation and positive psychological outcomes.

## 5. Conclusions

In summary, this study underscored the significance of arts participation in daily life. The research revealed that the psychological benefits of arts participation vary with forms of art and operationalization approaches employed; the diversity of arts engagement is associated with flow, aesthetic emotions, ego identity, and flourishing. It highlighted various potential pathways to achieve flourishing through arts participation. The findings support the perspective that the psychological benefits of arts participation are not confined to specific domains. This study contributes to a deeper understanding of pathways to a fulfilling life and offers valuable insights for the formulation of art education policies and art intervention programs. Future studies must further explore the psychological benefits of arts participation in the field of positive psychology.

## Figures and Tables

**Table 1:** Regression analysis of types of arts engagement on positive psychological outcomes.

Variable	Model 1			Model 2			Model 3			Model 4		
	Flow			Aesthetic Emotion			Ego Identity			Flourishing		
	*β*	*SE*	*t*	*β*	*SE*	*t*	*β*	*SE*	*t*	*β*	*SE*	*t*
Gender (male = 1)	−0.11	1.09	−2.32 *	−0.10	0.47	−2.17 *	−0.03	0.25	−0.53	−0.03	0.82	−0.64
Age	−0.03	0.33	−0.72	−0.12	0.14	−2.56 *	−0.04	0.07	−0.88	−0.07	0.25	−1.30
SFES	0.11	0.30	2.37 *	0.03	0.13	0.65	0.21	0.07	4.30 ***	0.20	0.22	3.99 ***
ALE (yes = 1)	0.24	1.23	5.02 ***	0.18	0.53	3.76 ***	−0.06	0.28	−1.14	0.01	0.93	0.17
VACR (yes = 1)	0.08	1.05	1.65	0.04	0.45	0.94	0.08	0.24	1.60	0.08	0.79	1.53
PACR (yes = 1)	0.09	1.17	1.99 *	0.12	0.50	2.63 **	0.03	0.26	0.64	0.04	0.87	0.75
LACR (yes = 1)	0.06	1.07	1.32	0.05	0.46	1.16	0.12	0.24	2.42 *	0.04	0.80	0.82
VACO (yes = 1)	0.07	1.08	1.54	0.14	0.46	2.83 **	0.04	0.24	0.73	0.01	0.81	0.08
PACO (yes = 1)	0.01	1.16	0.20	−0.06	0.50	−1.22	0.01	0.26	0.17	0.09	0.87	1.68
LACO (yes = 1)	0.02	1.34	0.50	0.08	0.58	1.78	0.01	0.30	0.10	−0.04	1.01	−0.90
*F*	8.94 ***			8.41 ***			3.88 ***			3.53 ***		
*R^2^*	0.18			0.17			0.09			0.08		

*Note.* SFES = Subjective family economic status; ALE = Art-learning experience; VACR = Visual arts creation; PACR = Performing arts creation; LACR = Literary arts creation; VACO = Visual arts consumption; VACO = Performing arts consumption; LACO = Literary arts consumption; * *p* < 0.05, ** *p* < 0.01, *** *p* < 0.001.

**Table 2:** Regression analysis of arts participation diversity and positive psychological outcomes.

Variable	Model 1			Model 2			Model 3			Model 4		
	Flow			Aesthetic Emotion			Ego Identity			Flourishing		
	*β*	*SE*	*t*	*β*	*SE*	*t*	*β*	*SE*	*t*	*β*	*SE*	*t*
Gender (male = 1)	−0.10	1.08	−2.23 *	−0.09	0.47	−1.99 *	−0.02	0.24	−0.50	−0.03	0.81	−0.63
Age	−0.04	0.32	−0.84	−0.13	0.14	−2.76 **	−0.06	0.07	−1.18	−0.06	0.24	−1.26
SFES	0.11	0.30	2.34 *	0.02	0.13	0.45	0.21	0.07	4.34 ***	0.2	0.22	4.12 ***
ALE (yes = 1)	0.25	1.21	5.36 ***	0.20	0.53	4.16 ***	−0.05	0.27	−1.01	0.01	0.91	0.21
APD	0.17	0.37	3.62 ***	0.19	0.16	4.12 ***	0.13	0.08	2.68 **	0.10	0.28	2.00 *
*F*	17.55 ***			14.98 ***			6.97 ***			6.34 ***		
*R^2^*	0.17			0.15			0.08			0.07		

*Note.* SFES = Subjective family economic status; ALE = Art-learning experience; APD = Arts participation diversity; * *p* < 0.05, ** *p* < 0.01, *** *p* < 0.001.

**Table 3:** Regression analysis of flow, aesthetic emotion, and ego identity between arts participation and flourishing.

Variable	Model 1			Model 2			Model 3			Model 4			Model 5		
	Flourishing			Flow			AE			EI			Flourishing		
	*β*	*SE*	*t*	*β*	*SE*	*t*	*β*	*SE*	*t*	*β*	*SE*	*t*	*β*	*SE*	*t*
Gender	−0.03	0.81	−0.63	−0.10	1.08	−2.23 *	−0.09	0.47	−1.99 *	0.001	0.24	0.02	0.02	0.64	0.43
Age	−0.06	0.24	−1.26	−0.04	0.32	−0.84	−0.13	0.14	−2.76 **	−0.05	0.07	−1.15	0.001	0.19	0.03
SFES	0.20	0.22	4.12 ***	0.11	0.30	2.34 *	0.02	0.13	0.45	0.18	0.06	3.79 ***	0.08	0.18	1.92
ALE	0.01	0.91	0.21	0.25	1.21	5.36 ***	0.20	0.53	4.16 ***	−0.11	0.27	−2.30 *	−0.04	0.75	−1.07
AP	0.10	0.28	1.99 *	0.17	0.37	3.62 ***	0.19	0.16	4.12 ***	0.09	0.08	1.92	−0.03	0.23	−0.86
Flow										0.31	0.01	5.62 ***	0.14	0.03	3.00 **
AE										−0.07	0.03	−1.35	0.22	0.07	4.92 ***
EI													0.50	0.13	12.58 ***
*F*	6.34 ***			17.55 ***			14.98 ***			10.12 ***			39.49 ***		
*R^2^*	0.07			0.17			0.15			0.14			0.43		

*Note.* SFES = Subjective family economic status; ALE = Art-learning experience; AP = Arts participation; AE = Aesthetic emotion; EI = Ego identity; * *p* < 0.05, ** *p* < 0.01, *** *p* < 0.001.

**Table 4:** Bootstrap tests on mediation effects.

Item	Effect	BootSE	BootLLCI	BootULCI
Overall indirect effect	0.76	0.18	0.42	1.13
X→M1→Y	0.14	0.07	0.03	0.3
X→M2→Y	0.24	0.08	0.1	0.43
X→M3→Y	0.27	0.15	−0.01	0.56
X→M1→M3→Y	0.15	0.05	0.06	0.27
X→M2→M3→Y	−0.04	0.03	−0.12	0.02

*Note.* X represents arts participation, M1 represents flow, M2 represents aesthetic emotion, M3 represents ego identity, Y represents flourishing, LLCI and ULCI represent the lower and upper limits of the confidence interval, respectively, and if the interval does not include 0, it indicates a significant result.

## Data Availability

The datasets used and analyzed during the current study are available from the corresponding authors upon reasonable request.
